# Unsupervised Segmentation of Greenhouse Plant Images Based on Statistical Method

**DOI:** 10.1038/s41598-018-22568-3

**Published:** 2018-03-13

**Authors:** Ping Zhang, Lihong Xu

**Affiliations:** College of Electronics and Information Engineering, Tongji University, Shanghai, China

## Abstract

Complicated image scene of the agricultural greenhouse plant images makes it very difficult to obtain precise manual labeling, leading to the hardship of getting the accurate training set of the conditional random field (CRF). Considering this problem, this paper proposed an unsupervised conditional random field image segmentation algorithm ULCRF (Unsupervised Learning Conditional Random Field), which can perform fast unsupervised segmentation of greenhouse plant images, and further the plant organs in the image, i.e. fruits, leaves and stems, are segmented. The main idea of this algorithm is to calculate the unary potential, namely the initial label of the Dense CRF, by the unsupervised learning model LDA (Latent Dirichlet Allocation). In view of the ever-changing image features at different stages of fruit growth, a multi-resolution ULCRF is proposed to improve the accuracy of image segmentation in the middle stage and late stage of the fruit growth. An image is down-sampled twice to obtain three layers of different resolution images, and the features of each layer are interrelated with each other. Experiment results show that the proposed method can segment greenhouse plant images in an unsupervised method automatically and obtain a high segmentation accuracy together with a high extraction precision of the fruit part.

## Introduction

The phenotypic information of greenhouse crop is an important property of plants, which has been applied to assist the production and processing of agricultural products in some research works^1–3^. To obtain high-throughput plant phenotypic information automatically has a significant meaning for synergistic analysis of genome-environment-phenotype^4–7^. During the period of fruit growth, it is very helpful to monitoring crop growth if the phenotypic information of fruits can be acquired automatically, since the phenotypic information can be used to estimate the yield and analyze the influence of environment to production. In the literature, the convolutional neural network (CNN) has been applied to agriculture. However, an urgent problem is that a large amount of reliable training data is needed to train it. For the analysis of greenhouse plants, it is an important process to get enough high-quality labelled images. In this regard, a well-segmented image of the plant can help labeling the image fast and accurately. Thereafter, the part of fruit image can be extracted to make further phenotypic analysis.

Till now, various image segmentation algorithms have been proposed in literature, among which the ones that can extract image features through statistical methods are important and practical scientific techniques. The characteristic of statistical approaches is to model the image in a statistical way. Each pixel in the image is viewed as the probability distribution of a variable. And the combination of pixels that has the maximum probability should be found from a statistical perspective.

As a popular conditional probability distribution model, the conditional random field (CRF)^8^ has been applied widely to some fields, such as image processing and pattern recognition^9,10^. To take advantage of global information of the observation field, CRF can avoid the error caused by improper modeling. Hence, an algorithm is more suitable for image segmentation if the fully connected CRF is utilized. Shotton J. *et al*.^11,12^ proposed a new approach to represent the features of image combined with boosting classifier. It optimized the unary potential of Dense CRF, and the precision of segmentation can be improved even when the number of categories of objects in the image is large. Moreover, the inference algorithm of Dense CRF was considered in refs^13,14^, and a more efficient way was provided to calculate the pairwise potential, thereby improving the efficiency and the image segmentation accuracy of the algorithm.

Since CRF is a supervised learning model, generally, its unary potential is obtained in supervised methods. It needs a high-quality training set containing a large amount of labeled images to learn related models of all kinds of objects. This is not realistic in the greenhouse problem. In the scene of greenhouse, light condition is very complex, and the leaves overlap each other to form shadow areas in the images. There are many indistinguishable regions in the greenhouse images. It is difficult to label the objects in the highlight or shadow areas accurately by hand. Given that some mistakes are contained in the training set, the models learned from it is not reliable enough. Thus, it has a negative impact on the accuracy of segmentation in CRF. For greenhouse problems, it is hard to obtain very reliable results by supervised methods. To this end, we take unsupervised methods into account in our study. Latent Dirichlet Allocation (LDA)^15^ is an unsupervised learning method in the domain of language models to identify hidden information in a large collection of documents or corpus^16^. It has been applied to solve the problems of computer vision widely^17–19^. The conception of bag of words^20,21^ conversed the information of pixels to visual words, which solved the problem of encoding words to get a better result of image classification and segmentation. Ref.^22^ proposed an algorithm called Spatial Latent Dirichlet Allocation (SLDA) to encode the spatial structure of visual words better. It designed the vision documents considering the spatial structure of image and got a better image segmentation result than that obtained by conducting LDA directly. Despite the promising potential of LDA for different segmentation tasks, it needs to generate a uniform random number during each iteration, leading to noises in the segmentation result. Furthermore, the generative model LDA shows the similarities of similar data. To some extent, it has a poor performance to reflect the difference between different objects in the image. We expect to consider both similarities and differences of objects in the images, hence we can get more complete image information in the process of segmentation.

In this paper, we combine the above two methods, namely CRF and LDA, and propose an unsupervised learning method to segment the greenhouse plant images. The segmentation result of LDA is used as the initial labels of CRF. At first, LDA is modeled with the features of pixels, and the pixels are clustered into some classes according to the maximum probability. Thus, LDA can get more reliable label information than manual labeling to obtain the training set in the process of greenhouse plant image segmentation. Meanwhile, this method takes advantage of CRF to reflect the differences between pixels of different classes. Therefore, the proposed method makes the supervised method and the unsupervised method complementary to each other. Experimental results showed that this unsupervised learning method can achieve a high accuracy of image segmentation.

## ULCRF

### Statistical Model

Before discussing the unsupervised learning method Unsupervised Learning Conditional Random Field (ULCRF), we introduce the statistical models related to this method briefly.

#### CRF (Conditional Random Field)

CRF calculates the conditional probability distribution $P(Y|X)$ of random variable **Y** (label sequence) given random variable **X** (observation sequence), which can be described as follow^9^:

1 $$P(Y|X)=\frac{1}{Z(X)}\tilde{P}(Y,X)$$

where $\tilde{P}(Y,X)=\exp {\sum }_{i}{{\omega }}_{i}\times {f}_{i}(Y,X)$, $Z(X)={\sum }_{Y}\exp (\sum _{i}{{\omega }}_{i}\times {f}_{i}(Y,X))$, ${f}_{i}(Y,X)$ represents the feature function, *ω*_*i*_ is the weight of the corresponding feature function. The CRF is a sum of every joint probability distribution of random variables **X** and **Y**.

For the problem of image segmentation, we establish a fully connected CRF. Suppose that the observation sequence *I*:{*I*_1_, …, *I*_*N*_} represents a set of input images, for which the label sequences are *X*:{*X*_1_, …, *X*_*N*_} that take their values in the domain of the set *L* = {*l*_1_, *l*_2_, …, *l*_*k*_}. The Gibbs distribution of CRF can be described as follow:

2 $$P(X|I)=\frac{1}{Z(I)}\exp (-{\sum }_{c\in {{C}}_{{G}}}{\varphi }({X}_{c}|I))$$

The corresponding Gibbs Energy is

3 $$E(x)=\sum {{\psi }}_{u}({x}_{i})+\sum {{\psi }}_{p}({x}_{i},{x}_{j})$$

where *i* and *j* take values from 1 to *N*. The unary potential ${{\psi }}_{u}({x}_{i})$ is computed independently for each pixel by a classifier that produces a distribution over the label assignment *x*_*i*_ given image features. The pairwise potential ${{\psi }}_{p}({x}_{i},{x}_{j})$ is computed in the correlation of pixels to identify the category information of each pixel. Afterwards, we determine the label assigned to each pixel by computing the probability distribution.

#### LDA (Latent Dirichlet Allocation)

**Figure 1:**
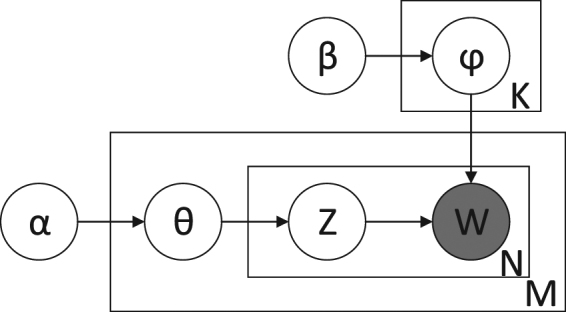
The graphical model of LDA.

It is known that the generative probabilistic model LDA can be applied to calculate the topic probability of words in documents. The basic idea of this model is to view documents as random mixtures over latent topics, where each topic is characterized by a distribution over words. Its graphical model is shown in Fig. 1. A document consists of a sequence of *N* words denoted by *W* = (*w*_1_, *w*_2_, …, *w*_*N*_), and a corpus is a collection of *M* documents. All the words in a corpus will be clustered into *K* topics, where each one is modeled as a multinomial distribution over the codebook. Suppose that *α* and *β* are Dirichlet prior hyper parameters. A multinomial parameter *θ* over the *K* topics is sampled from Dirichlet prior as *θ* ~ *Dir*(*α*). Topic **z** is the multinomial distribution of *θ*:*z* ~ *Multinomial*(*θ*). For a topic *k*, the polynomial parameter *φ*_*k*_ is sampled from the Dirichlet prior such that *φ*_*k*_ ~ *Dir*(*β*). The value **w** of a word is sampled from the discrete distribution of topic **z**:*w* ~ *Discrete*(*φ*_*z*_).

The joint probability distribution of the model shown in Fig. 1 is:

4 $$P(Z,W,{\theta },\phi |{\alpha },{\beta })=P(W|{\phi },Z)\cdot P(Z|{\theta })\cdot P(Z)\cdot P({\phi })$$

where, the parameters *θ*, *φ*, *α*, *β* have been described above. For the sake of simplicity, we can simplify the Equation (4) as follows:

5 $$P(Z,W)=P(W|{\phi },Z)\cdot P(Z|{\theta })\cdot P(Z)\cdot P({\phi })$$

The probability of each topic is iteratively calculated by Gibbs sampling. Thereafter, the visual words are clustered into topics which correspond to object classes.

### Unsupervised Learning CRF

The joint probability distributions $\tilde{{P}}({Y},{X})$ and ${Z}({X})$ in Eq. (1) are obtained by learning a mass of samples from the training set. At the beginning, we took some tomato plant images from a greenhouse to label them manually. However, some serious problems should be addressed during the labeling process. It is ubiquitous that all kinds of objects reflect light and the leaves overlap each other, which makes the objects under reflective or shadow areas to be different from normal ones in appearance. Sometimes, it is impossible to confirm exactly what the objects in these areas are. There are also some objects far away from the lens, causing difficulties in labeling them. Under these conditions, the manual labeled training set is not accurate enough.

As an unsupervised learning method, LDA delves the individual information of pixels to get the joint probability distribution of pixels and classes. Each pixel in the image is represented by a feature vector. For the components of plant image which are difficult to distinguish manually, their dissimilarities can be reflected by calculating the probabilities of these vectors. Hence, the distributions $\tilde{{P}}({Y},{X})$ and ${Z}({X})$ in Eq. (1) that should be learned from the training set, can be calculated by the joint probability distribution *P*(*Z*, *W*) in LDA. For eq. (3), the unary potential *ψ*_*u*_ of Gibbs Energy shows the individual information of pixels. It is computed by a classifier as described in Section 2.1.1, and the LDA can fit its role. We can apply the unsupervised learning method LDA to get the unary potential of CRF. It avoids negative influence of the unreliable greenhouse plant image training set of the supervised learning method. As for the second term *ψ*_*p*_(*x*_*i*_, *x*_*j*_) in Eq. (3), the pairwise potential categorizes the pixels depending on the inter-pixel relationships, which is outside the scope of this study. A highly efficient inference algorithm based on a mean field approximation to the CRF distribution^14^ is applied here.

There are some noises in the segmentation results of LDA due to the generation of random number in the iteration process. These noises can be removed in the subsequent calculation of pairwise potential for CRF. To some extent, CRF avoids noise generation, one of LDA’s disadvantages. These two algorithms can be complementary to each other. The method described in this section can be summarized as follows: Firstly, the initial labeling of the plant image is calculated by LDA. Then the initial segmentation determined from the individual features of pixels is viewed as unary potential of CRF. Finally, a mean field approximation is applied to obtain the pairwise potential of CRF, and ensure the class of each pixel. Since the unary potential is calculated by LDA, the training process of CRF is replaced by an unsupervised learning method. We name the algorithm described in this section Unsupervised Learning CRF, abbreviated as ULCRF.

## MR-ULCRF Method

Usually, the color, shape and density of greenhouse crops are changeable at different cultivation periods. As a result, the features of greenhouse plant images are also different at these periods. It may not be reasonable to segment plant images with a fixed scale throughout the whole period of crops. To cope with this problem, we can take advantage of the image multi-resolution modeling. It is known that the resolution is an important property of images. For instance, it is difficult to observe some features at a specific resolution, while they can be reflected at another resolution. Here, we take the greenhouse plant image as an example: when the image has high resolution, pixels in a window of specific size in the image may be part of a leaf or a fruit; however, at low resolution, pixels within a window of the same size may be the image of a complete leaf or a fruit. In these two resolutions, we can extract different information from the same size of image window^23–28^. Therefore, we can mine richer image information based on multi-resolution modeling.

Some factors such as glasses, plastic films and pipelines may reflect light in the greenhouse. As a result, there is evident light reflection on the surface of the leaves and fruits, leading to highlight regions on the image. In this regard, the features of objects that reflect light cannot be sufficiently well described. On the other hand, the pixels of shadow areas appear to be dark colors, which are different from those on non-shaded areas. Thus, it is inevitable to make mistakes in segmentation of these objects. Note that, some small highlight or shadow areas become smaller when reducing the resolution of the image, resulting in the reduction of feature differences between the same objects. Therefore, the negative impacts on the segmentation result will be mitigated. After obtaining the segmentation result of low resolution, we map it to a high-resolution image, therefore reducing the misclassification of highlight and shadow areas.

In this article, considering the size of images, we can down-sample an image twice to produce three layers of different resolutions, where the top layer has the lowest image resolution. For the feature association between each layer, since a more accurate annotation can lead to a more precise segmentation result of CRF, the segmentation result of the upper layer image (lower resolution) is used as the annotation of the lower layer image (higher resolution). Note that, the image is blurred if the resolution of the image is reduced, and hence, the influence of noise on the image is diminished. Here, we present the process of multi-resolution image segmentation briefly. For the top layer image (lowest resolution), the method described in Section 2.2 is adopted to get an initial segmentation by LDA. After that, the initial segmentation is viewed as the unary potential for CRF to further obtain the final segmentation result of this layer of image. By using the above-mentioned method of associating two layers of images, we map the segmentation of the low-resolution image to the high-resolution image, obtaining the segmentation result of the image with the original resolution. We name this unsupervised CRF on multi-resolution images as multi-resolution ULCRF, abbreviated as MR-ULCRF.

## Experiments

In our research, all the images were taken under real field conditions from the glass greenhouses of the Sunqiao Modern Agricultural Development Zone in Shanghai and the Chongming Base of National Facility Agricultural Engineering Technology Research Center. It deserves pointing out that, all the ground truths and training set for comparison experiments were labeled manually by the author. We consider the images of tomatoes, which have a resolution of 200 × 300. All experiments were conducted on a 1.40 GHz machine with 6GB memory.

### Visual word and document definition of LDA

A local descriptor is computed for each image patch and quantized into a visual word. To obtain local descriptors, images are convolved with the filter bank proposed in^21^, which has shown to have good performance for object categorization. After that, each pixel is represented as a feature vector, namely the descriptor. We divide an image into local patches on a grid and densely sample a local descriptor for each patch. The K-means algorithm is used to cluster these local descriptors in the image into a code book of size *W*. Next, these visual words are clustered into classes.

**Figure 2:**
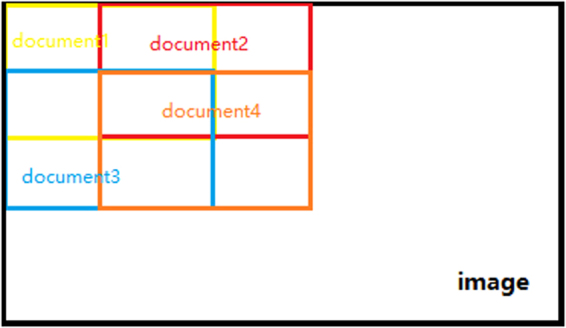
The overlapped document assignment.

According to ref.^22^, we cannot get good segmentation result to view an image as a single document, because there will be a lot of noise in the segmentation result. It is known that, if visual words are from the same class of objects, they not only often co-occur in the same image but are also close in space. Therefore, an image should be divided into several documents, and the image patches that are close in space should be grouped into the same document. A straightforward method is to divide an image to several regions equally on a grid, where each region is viewed as a document. However, we may divide pixels belonging to the same object into two regions (documents) in the process of grid division, which cause misclassification to some extent. To solve this problem, we put many overlapped regions on the image, each of which is a document. Hence, there will always be some regions containing almost all the pixels of an object in the image. The overlapped document assignment is shown in Fig. 2.

### The extraction of foreground fruit image

For greenhouse plant images, objects can generally be divided into three classes: fruits, leaves and backgrounds. However, as the nature of unsupervised learning, both ULCRF and MR-ULCRF can only segment different classes of objects but cannot point out the specific name of each class. After getting the segmentation results, we developed a strategy to determine the name (fruit, leaf, background) of each class. Through analyzing the color feature of each class on greenhouse images, we found that the main color of fruits part tends to be red, while that of leaves part tends to be green, and the color of the other background objects in greenhouse tends to be bright white. For the pixels belonging to each class, we firstly calculate the mean value of each color component of RGB, from which the variance of these three mean values is calculated. The background class has the minimum variance. For the remain two classes of fruit and leaf, the mean value of the R component of fruit class is greater than that of the other two color components, and the mean value of the G component of leaf class is greater than that of the other two color components. Through the above calculation, we can determine the specific name of each class on the greenhouse images. Then we can extract the fruit part from the image easily.

### The experimental results of ULCRF

**Figure 3:**
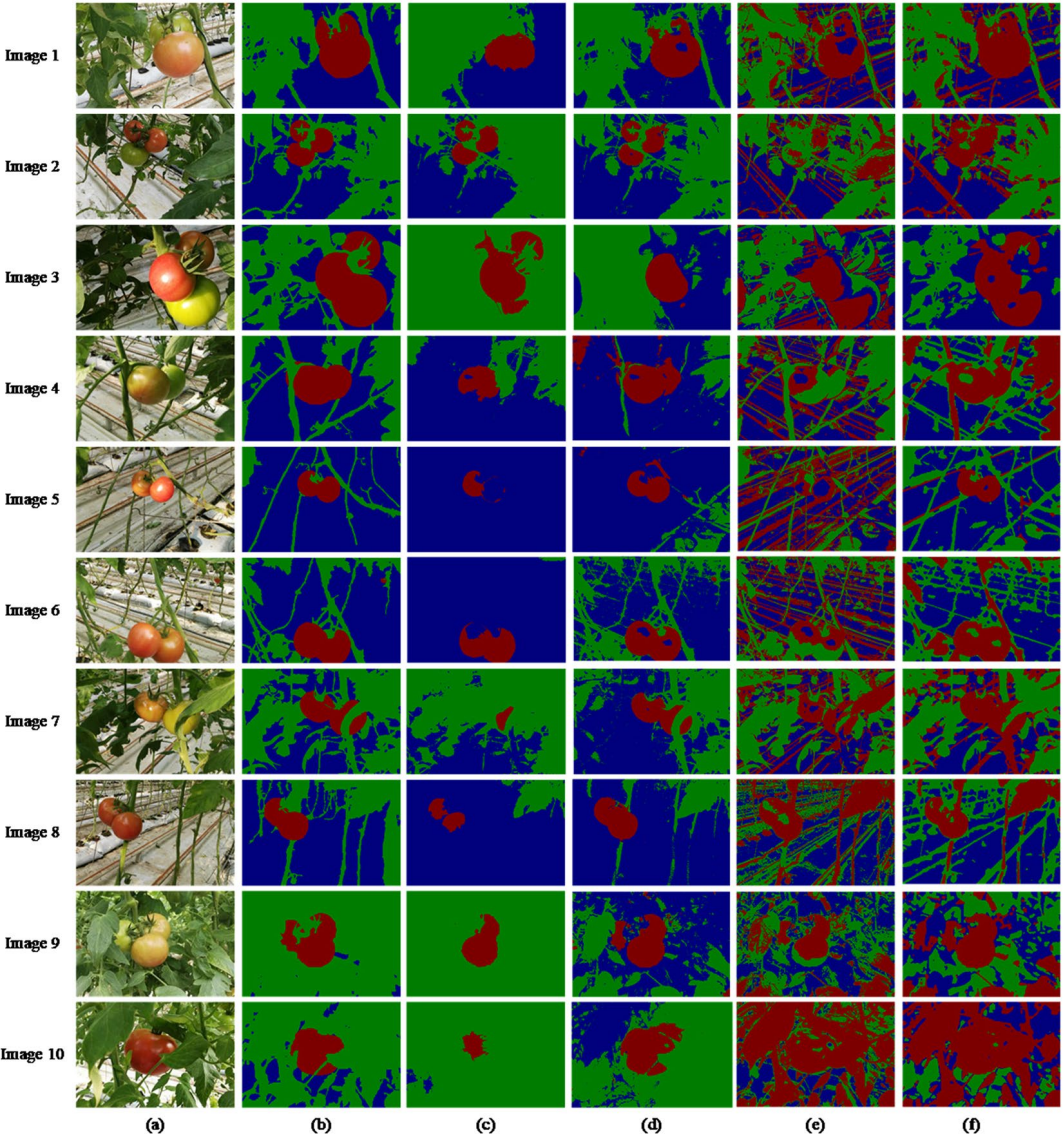
Comparison experiment of image segmentation: (**a**) the original greenhouse plant images; (**b**) the ground truths of segmentation; (**c**) Texton Boost; (**d**) ULCRF; (**e**) OTSU; (**f**) MRMRF.

In this section, we show experimental results of the ULCRF method. In ref.^11^, a supervised learning method, namely Texton Boost, was applied to calculate the unary potential of CRF. To compare the image segmentation qualities between supervised and unsupervised learning method, we have a contrast experiment between the ULCRF and Texton Boost. Meanwhile, there are two other common image segmentation methods used for contrast experiments. They are the OTSU method and the Multi-resolution Markov Random Field (MRMRF) in the wavelet domain. The comparison of the segmentation results is shown in Fig. 3.

**Figure 4:**
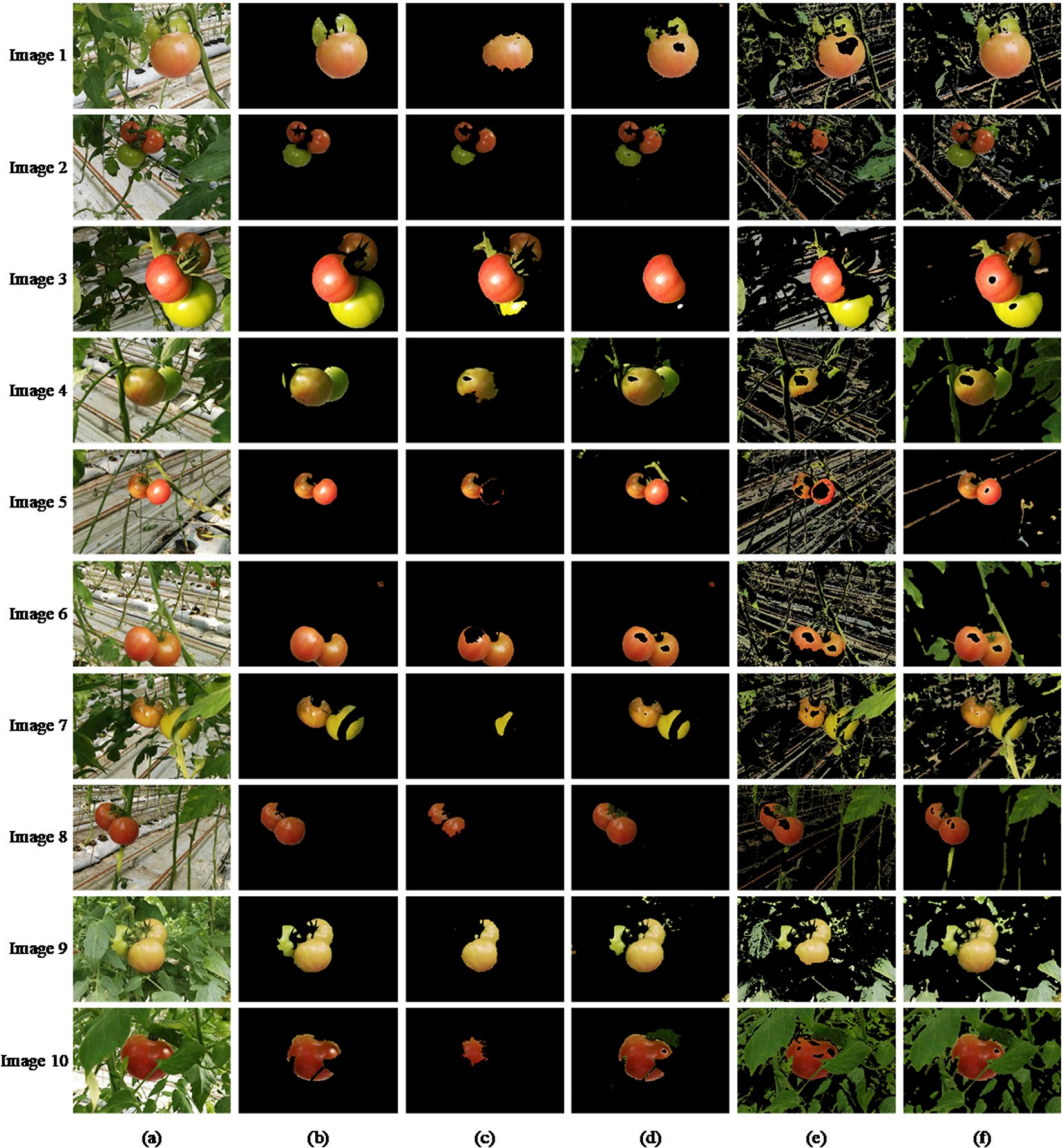
Comparison of fruit image segmentation results: (**a**) the original images; (**b**) the ground truths of fruit images; (**c**) Texton Boost; (**d**) ULCRF; (**e**) OTSU; (**f**) MRMRF.

As described in Section 4.2, after getting the segmentation results of the original images, we keep the fruit part pixels on the image and set RGB values of other part pixels to be zero to extract the image of fruits. Figure 4 shows the fruit image segmentation results of the same original images with Fig. 3.

To demonstrate and compare the segmentation qualities of these methods more apparently, we calculate the accuracy of image segmentation and the fruit image segmentation respectively. The accuracy of image segmentation is defined as:

6 $$Ac{c}_{seg}=\frac{k}{(m\times n)}$$

where *k* is the number of pixels that have the same label as the ground truth, *m* and *n* are the width and height of the image. The unit of measure is pixel. In other words, *m* × *n* is the number of pixels on the image.

The accuracy of fruit image segmentation is defined as:

7 $$Ac{c}_{fruit}=\frac{{l}_{fruit}}{a}$$

where *l*_*fruit*_ is the number of pixels which have the same fruit label as the ground truth, *a* is the total number of pixels labeled as fruit on the ground truth. The comparison of calculated image segmentation accuracy is shown in Table 1.

**Table 1:** The calculated image segmentation accuracy of comparison experiments.

	Image 1	Image 2	Image 3	Image 4	Image 5
Texton Boost	0.8040	0.9071	0.5755	0.7616	0.8499
ULCRF	**0**.**9409**	**0**.**9367**	0.5223	**0**.**7672**	0.8132
OTSU	0.7217	0.7335	0.6154	0.6569	0.4818
MRMRF	0.8236	0.8004	0.7835	0.5517	0.7811
	**Image 6**	**Image 7**	**Image 8**	**Image 9**	**Image 10**
Texton Boost	0.7267	0.7376	0.7359	0.9577	0.7876
ULCRF	**0**.**8890**	**0**.**7676**	**0**.**8254**	0.4272	0.6178
OTSU	0.5617	0.7064	0.4401	0.4309	0.3516
MRMRF	0.6680	0.7867	0.6184	0.4899	0.1408

The comparison of the fruit segmentation accuracy is shown in Table 2.

**Table 2:** The fruit segmentation accuracy of comparison experiments.

	Image 1	Image 2	Image 3	Image 4	Image 5
Texton Boost	0.5780	0.7126	0.5911	0.4971	0.4673
ULCRF	**0**.**8158**	**0**.**8859**	0.3694	**0**.**9277**	**0**.**9824**
OTSU	0.7758	0.4596	0.5249	0.3701	0.5777
MRMRF	0.9803	0.9960	0.8347	0.8170	0.9828
	**Image 6**	**Image 7**	**Image 8**	**Image 9**	**Image 10**
Texton Boost	0.8059	0.1512	0.5187	0.6956	0.2518
ULCRF	**0**.**9034**	**0**.**8543**	**0**.**9657**	**0**.**9511**	**0**.**9736**
OTSU	0.4812	0.8119	0.8211	0.7210	0.8100
MRMRF	0.9195	0.9961	0.9087	0.9907	0.9792

**Figure 5:**
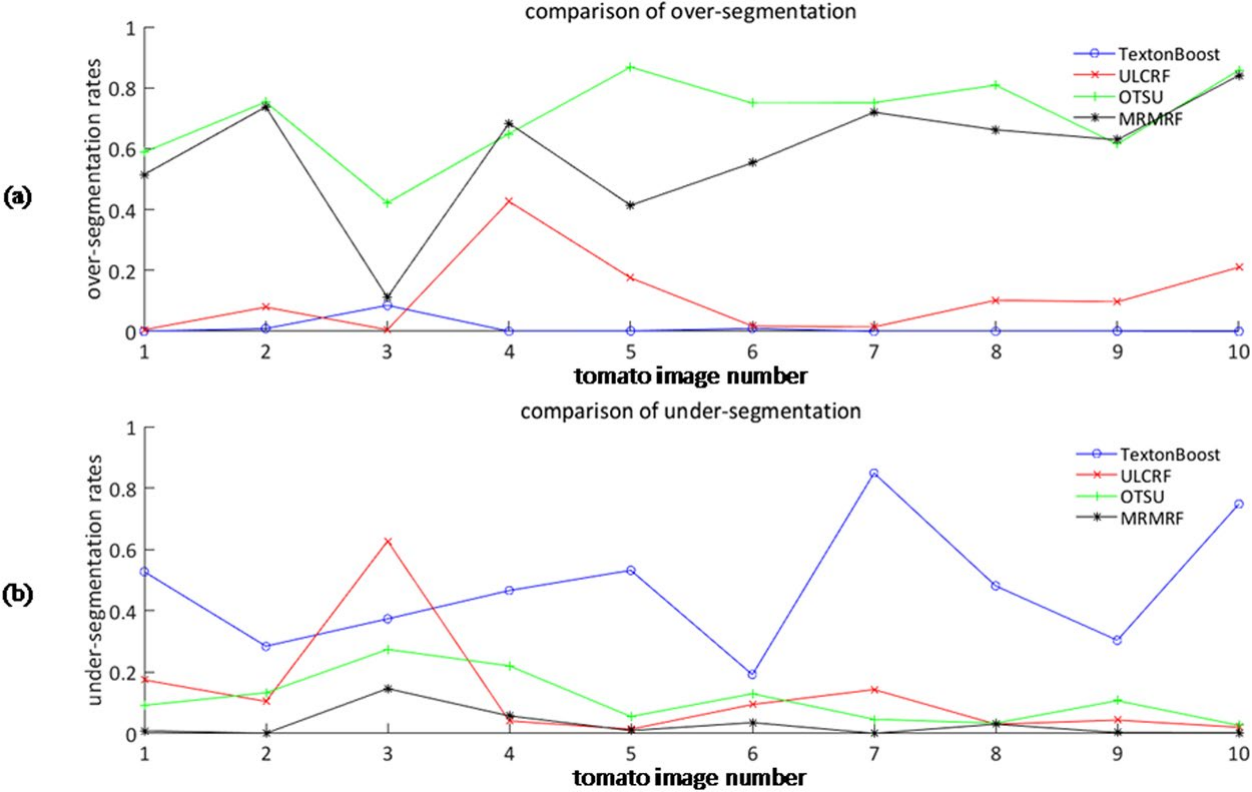
Segmentation rates of fruit images: (**a**) over-segmentation rate; (**b**) under-segmentation rate.

Since our goal is to obtain the image information of fruits, we calculate the over-segmentation rate and under-segmentation rate of the fruit image to further compare the above methods. The rate of over-segmentation and under-segmentation are, respectively, defined as follows:

8 $$Se{g}_{over}=\frac{{P}_{over}}{{P}_{gt}+{P}_{over}}$$

9 $$Se{g}_{under}=\frac{{P}_{under}}{{P}_{gt}+{P}_{over}}$$

where *P*_*gt*_ is the number of fruit pixels in the fruit image ground truth, *P*_*over*_ is the number of fruit pixels that exist in the fruit image segmentation result but do not exist in the fruit image ground truth, *P*_*under*_ is the number of fruit pixels that should but do not exist in the fruit segmentation result. We draw line charts of the over-segmentation rates and the under-segmentation rates of fruit image of the above four segmentation methods in Fig. 5.

From the comparison of segmentation results, the accuracy, and the rates of segmentation, the ULCRF method is superior to the supervised learning method to some extent. Here we first analyze the results of comparison experiments. In the process of labeling training set for Texton Boost manually, almost on every image, there are some regions locate in the shadow of leaves or highlight areas. There are also some objects far away from the lens. We cannot exactly determine what they are at all. Note that, the number of labeled images suitable for the training set is limited, and the training set is not very accurate. Hence, the calculation of both unary potential and pairwise potential of CRF are adversely affected. In addition, pixels with similar characteristics may represent different objects on different images, hence the different labels assigned. The supervised learning method cannot obtain a model with high recognition of these pixels. For example, the characteristics of unripe fruits and leaves are similar. However, it can be particularly observed that, for the fruits of high under-segmentation rate of Texton Boost, some are misclassified as leaves or backgrounds. Therefore, in cases of complex greenhouse scene, the probability distribution obtained through this supervised learning method is not accurate enough.

**Figure 6:**
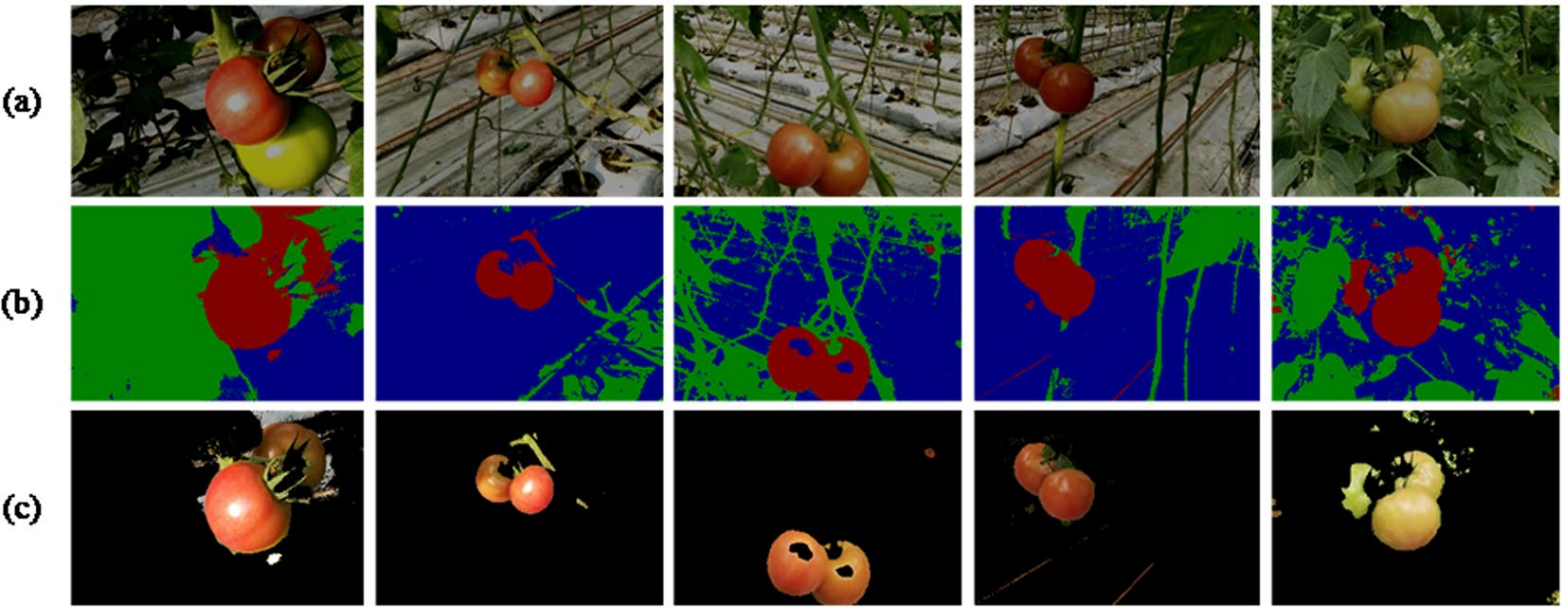
Segmentation results of adjusted images: (**a**) simulated images under shade screen; (**b**) image segmentation results; (**c**) fruit segmentation results.

Although it is impossible for LDA to label every pixel precisely, the statistical method that cluster every pixel in the aspect of feature vectors can get a relatively reliable initial labeling result. Subsequently, a more precise segmentation can be obtained through the mean field approximation. It deserves noticing that, we can only extract one of the three fruits on the image for the image 3, both the accuracy of image segmentation and that of fruit segmentation are not satisfactory. In this image, the difference of light reflection between each fruit is quite large, and there is prominent feature difference between them, which affects the feature clustering and the correct calculation of the probability distribution of LDA. For this kind of images, the accuracy of later image segmentation can be improved through a simple preprocessing step or a more reasonable way of image collection, such as taking images under a shade screen to reduce reflection. Here we take the image 3 under a shade screen in simulation through adjusting the intensity, saturation and contrast of this image. Figure 6 shows the segmentation results of adjusted images. We select some other images with the same adjustment as contrasts.

Compared with the previous segmentation result in Table 1 and Table 2, the accuracy of image and fruit segmentation increased to 0.5537 and 0.5776 respectively for image 3, which is similar to other contrast methods. For other comparison images, the segmentation results are still satisfactory. From the result of fruit segmentation, all the ripe fruits have been segmented, which has met the requirement of dynamic yield estimation. We can assume that, images taken under a true shade screen should have a much lower level of reflection than the ones we simulated. And differences between several fruits will also be smaller. It is credible that our ULCRF can perform better in that circumstance. Since the shade screen is an essential facility to diminish the radiation in greenhouse, it is feasible to take images under it. Thus our method has an advantage as it is applicable in segmenting greenhouse images.

From the running time, the average execution time for ULCRF is 95.45 s. For Texton Boost, the training procedure takes 50 minutes for 700 rounds on the training set of 45 images. The average execution time for Texton Boost to segment an image is 125.07 s. Therefore, the supervised learning method has no advantage in running time.

For the other two contrast experiments of the OTSU and the MRMRF, it is obvious that these two methods are not applicable in segmenting the greenhouse plant images. The OTSU method segment images into a few classes through setting thresholds. Obviously, it is not suitable to process the complex plant images only through setting thresholds. As for the MRMRF, the features are obtained by wavelet transform on the RGB components of pixels. Although the wavelet transform was carried out under multiresolution condition to get more features from the images, it is not enough to describe the complicated greenhouse plant features merely based on the RGB color components of the image. Our feature vectors described in Section 4.1 have shown the advantages here.

Through qualitatively and quantitatively analyzing comparison experiments, ULCRF is an efficient way to segment greenhouse plant images in terms of the quality of training set and running time.

### Multi-resolution modeling and image pyramid

**Figure 7:**
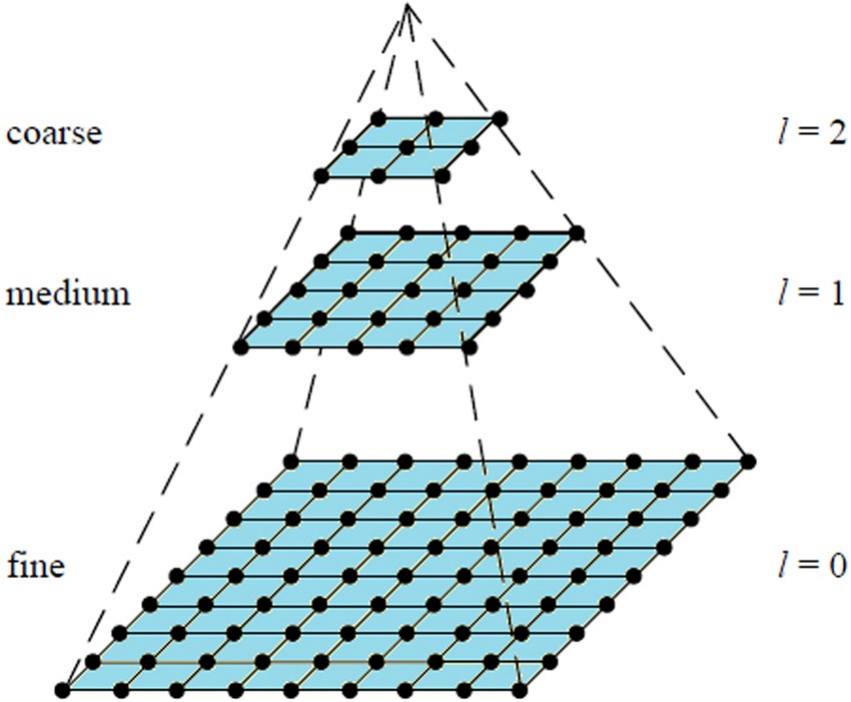
The structure of the image pyramid.

As described in Section 3, we generated an image pyramid to obtain more image features and reflect the diversity of features in different cultivation periods. The original image consisting of 200 × 300 pixels is down-sampled twice in the *x* and *y* directions to get two layers of images, their sizes are 100 × 150 pixels and 50 × 75 pixels respectively. Each layer of image is convolved with the filter bank mentioned in Section 4.1 to obtain the feature expression. The structure of image pyramid is shown in Fig. 7.

After obtaining the image pyramid, the image segmentation process is carried out through the MR-ULCRF described in Section 3. We can obtain the segmentation result of the image at the bottom layer (original image).

### The experiment results of MR-ULCRF

**Figure 8:**
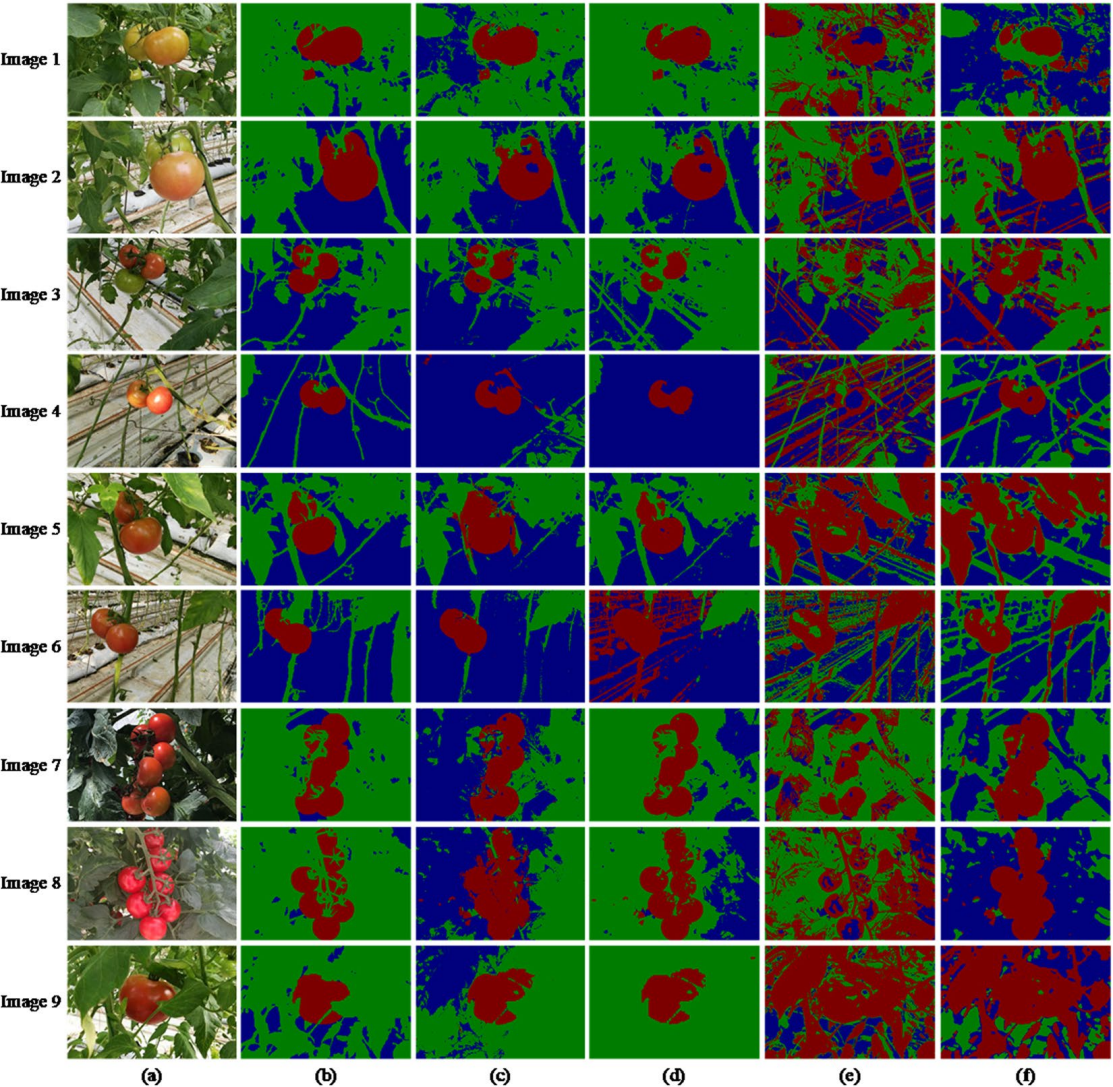
Contrast experiment of the image segmentation: (**a**) the original images; (**b**) the ground truths; (**c**) ULCRF; (**d**) MR-ULCRF; (**e**) OTSU; (**f**) MRMRF.

In this section, we show the segmentation results of the MR-ULCRF method. In contrast, we perform the ULCRF approach on single-layer images to get the segmentation results. Moreover, we employ the other methods (e.g., OTSU and MRMRF) for the purpose of comparison. The experiment results obtained by all these approaches are shown in Fig. 8.

**Figure 9:**
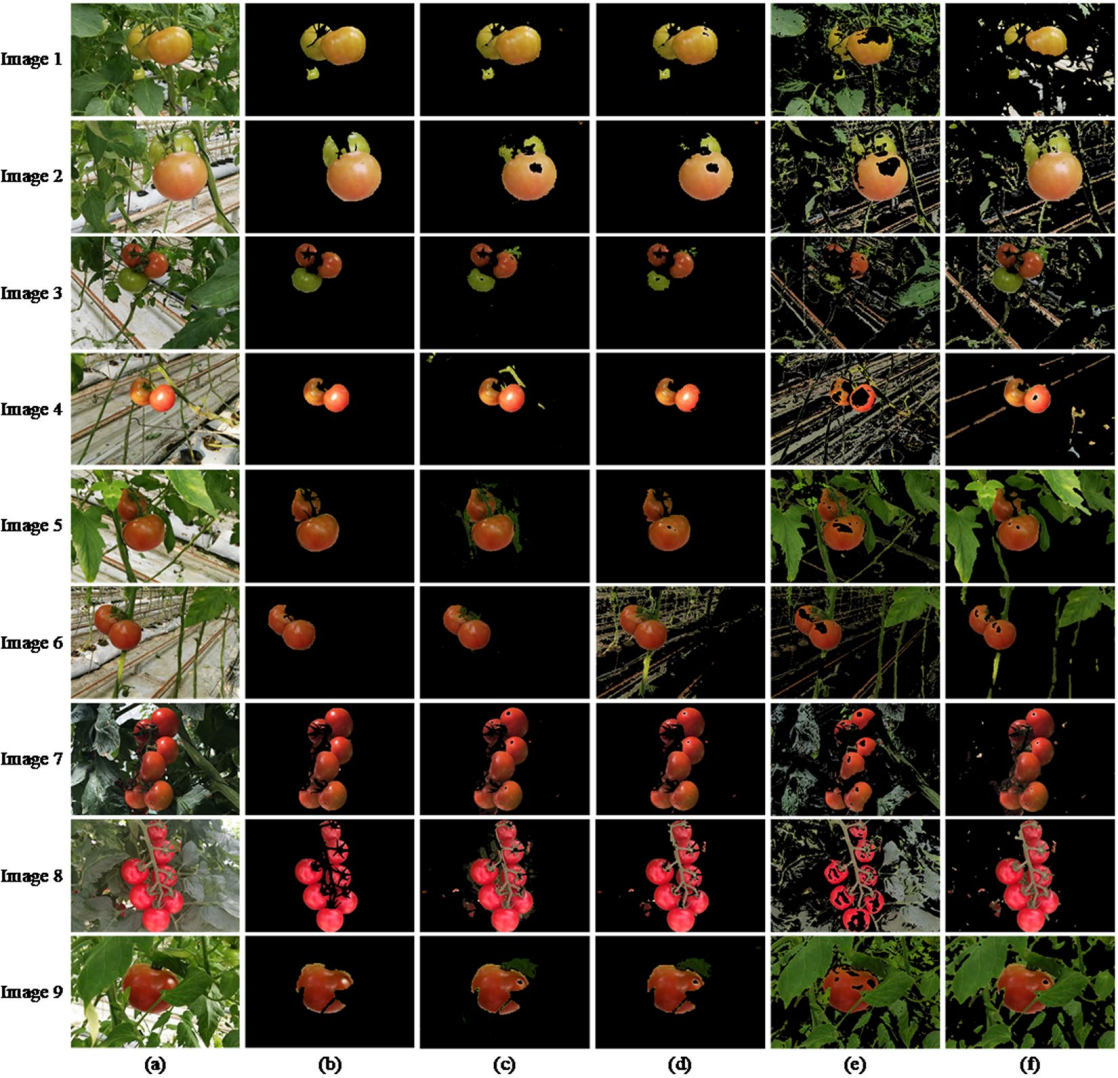
Comparison of fruit image segmentation results: (**a**) original images; (**b**) ground truths of fruit images; (**c**) ULCRF; (**d**) MR-ULCRF; (**e**) OTSU; (**f**) MRMRF.

After getting the segmentation of original images, we extract the component of fruits in each image. The segmentation results of fruit images are shown in Fig. 9.

We calculated the image segmentation accuracy, the fruit image segmentation accuracy, and the fruit over-segmentation and under-segmentation rates described in Section 4.3. Table 3 shows the comparison of image segmentation accuracy on these methods.

**Table 3:** Comparison of image segmentation accuracy.

	Image 1	Image 2	Image 3	Image 4	Image 5
ULCRF	0.6980	**0**.**9409**	**0**.**9367**	0.8132	0.9244
MR-ULCRF	**0**.**9597**	0.9300	0.8136	**0**.**8949**	**0**.**9669**
OTSU	0.6526	0.7217	0.7335	0.4818	0.4656
MRMRF	0.3123	0.8236	0.8004	0.7811	0.4896
	**Image 6**	**Image 7**	**Image 8**	**Image 9**	
ULCRF	**0**.**8254**	0.5631	0.4685	0.6178	
MR-ULCRF	0.6577	**0**.**7936**	**0**.**7447**	**0**.**8330**	
OTSU	0.4401	0.7094	0.5481	0.3516	
MRMRF	0.6184	0.6826	0.2545	0.1408	

The comparison of fruit segmentation accuracy is shown in Table 4.

**Table 4:** Comparison of fruit segmentation accuracy.

	Image 1	Image 2	Image 3	Image 4	Image 5
ULCRF	0.9674	0.8241	0.8859	0.9824	0.9889
MR-ULCRF	0.9419	0.7706	0.7797	0.9062	0.9497
OTSU	0.7004	0.7758	0.4596	0.5777	0.8967
MRMRF	0.6158	0.9803	0.9960	0.9828	0.9598
	**Image 6**	**Image 7**	**Image 8**	**Image 9**	
ULCRF	0.9657	0.9817	0.9998	0.9736	
MR-ULCRF	0.9962	0.9493	0.9993	0.9547	
OTSU	0.8211	0.6554	0.5850	0.8100	
MRMRF	0.9087	0.9944	0.9996	0.9792	

**Figure 10:**
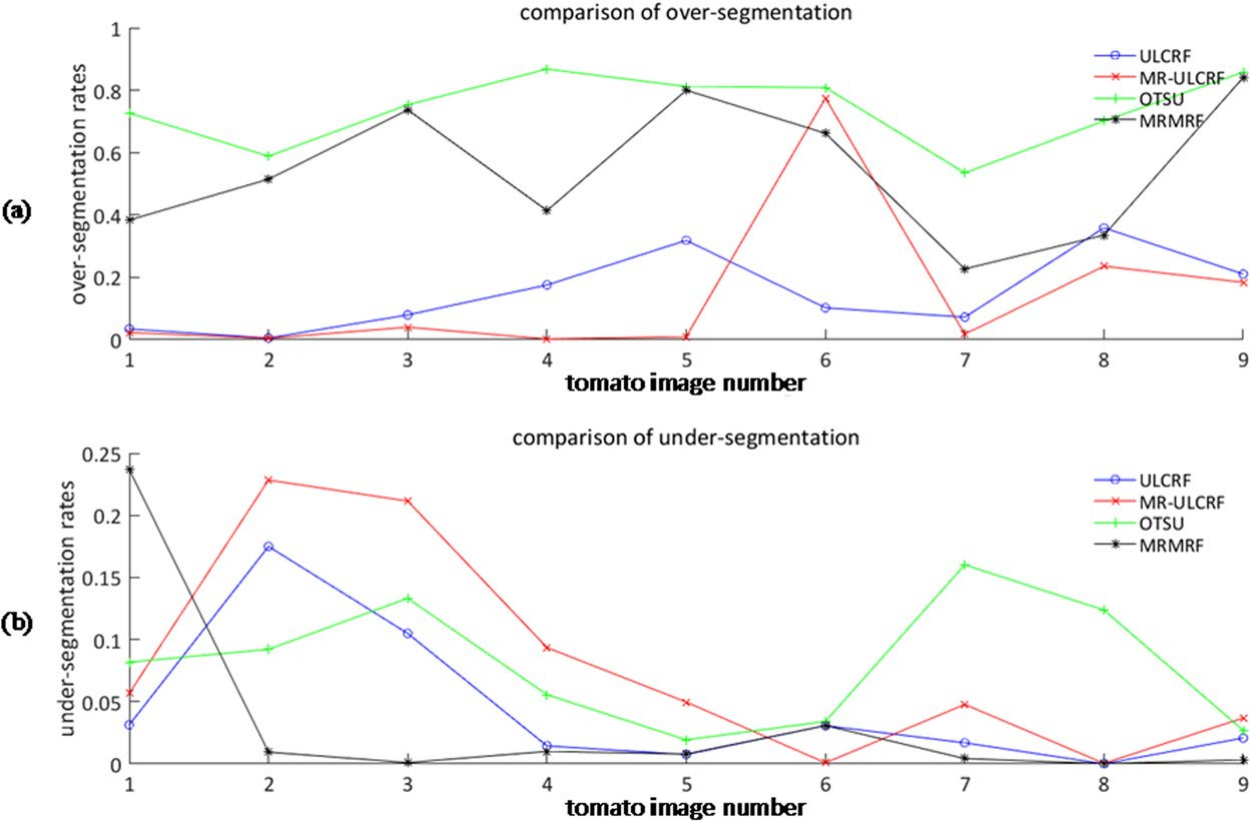
Segmentation rates of fruit images: (**a**) over-segmentation rates; (**b**) under-segmentation rates.

The line charts of over-segmentation and under-segmentation rates are shown in Fig. 10.

The average total execution time for ULCRF is 94.45 s, while it is 64.89 s for MR-ULCRF. For multi-resolution method, the processing speed of low resolution images is faster, and the total running time is less. To compare the segmentation results, the accuracy, and the segmentation rates, there is little difference between the results of fruit image segmentation obtained by methods ULCRF and MR-ULCRF. But there are some differences in the segmentation accuracy of the entire image. For the MR-ULCRF, the segmentation results of the upper layer image have a significant influence on the segmentation results of the next layer, and the final results are influenced through iterating segmentation result layer by layer. Before fruits ripening, they distribute loosely, or the number of these fruits is small. Also, some of them are green or not red enough. When the resolution is reduced, the differences between fruits and other objects are not obvious. Thus, the segmentation results of ULCRF is a little better than the MR-ULCRF under these circumstances. For example, in the image 2, image 3 and image 6, the MR-ULCRF have mislabeled part of the green fruits to the class of leaves, or mislabeled pipelines and stems to the class of fruits, their over-segmentation or under-segmentation rates are also a little higher. This is because the differences between unripen fruits and leaves or some other facilities are not obvious in the low-resolution image. It is observed that some cases of mislabeling occur on the initial scale of the image pyramid, resulting in the decrease of the final image segmentation accuracy. Note that, the fruits occupy more regions on the image, and they appear redder in the middle and the late periods of fruit growth. In these periods, the main cause of mislabeling is the highlight and shadow areas on the image due to the uneven illumination and light reflection. It can be well solved in a low-resolution image, thanks to the insensitive recognition of the objects with unobvious feature differences. For example, in the image 1 and image 5, the segmentation results of all kinds of objects obtained by MR-ULCRF have almost no difference from the ground truths. This method also shows better performance on segmenting the same class of objects with large difference of distance to the lens, such as the segmentation results of image 1, image 7 and image 8. Because for the same objects with different distances to the lens, the difference of their features is smaller than that with other objects in a low-resolution image. For these kinds of pictures, the MR-ULCRF can improve the accuracy of image segmentation.

For the approaches OTSU and MRMRF, the segmentation results obtained are still not satisfying. Since their shortcomings have been discussed in Section 4.2, we do not describe more here.

According to the above analyses, we can conclude that, at the early stage of growth, fruits are not red enough and distribute loosely. The single-layer image segmentation method ULCRF can obtain more accurate segmentation results for the greenhouse plant images. However, as fruits mature gradually and distribute closely at the middle and late fruit period, the MR-ULCRF can segment images with a high accuracy.

## Conclusions

In this study, we proposed a modified statistical model of CRF, namely ULCRF, to segment greenhouse plant images. Through our experiments in different cases, some conclusions are drawn as follows.

1. Commonly, there are many highlight and shadow areas on plant images, and some of the regions on the images cannot be distinguished accurately, which cause difficulties in analyzing these images. For example, supervised learning from the inaccurate labeled images of training set leads to a model with low recognition. In view of these complicated scenes of plant image in the greenhouse, we apply the unsupervised learning topic model LDA to calculate the unary potential as the initial label of CRF. The initial clustering of image features is carried out by the probability statistical model. And a more preferable rough classification result is obtained than that of manual labeling training. Through the Dense CRF algorithm, we can obtain a more precise segmentation result of the image. Experiments show that this method can obtain a better segmentation result than the supervised learning method.
2. At different cultivation period, fruits have different colors, shapes and distribution densities. As the fruits grow gradually, they are more distinct from other objects in some periods. As a result, the misclassification is mainly caused by interference of highlight and shadow regions, which result in the differences between the same objects in the greenhouse. In these regards, we propose a multi-resolution image segmentation method. Since the image feature information is obtained at different resolutions, it is hard to distinguish the same kind of objects with some feature differences in the original image of low-resolution. Thus, these objects will not be divided into different categories, which can reduce the possibility of mislabeling. The proposed method can improve the image segmentation accuracy to a certain extent in the case of a dense and lush distribution of fruits on the image.
